# Transcatheter Versus Surgical Aortic Valve Replacement in Low-Risk Patients for the Treatment of Severe Aortic Stenosis

**DOI:** 10.3390/jcm9020439

**Published:** 2020-02-06

**Authors:** Alberto Polimeni, Sabato Sorrentino, Salvatore De Rosa, Carmen Spaccarotella, Annalisa Mongiardo, Jolanda Sabatino, Ciro Indolfi

**Affiliations:** 1 Division of Cardiology, Department of Medical and Surgical Sciences, “Magna Graecia” University, 88100 Catanzaro, Italy; polimeni@unicz.it (A.P.); sabatosorrentino@hotmail.com (S.S.); saderosa@unicz.it (S.D.R.); spaccarorella@unicz.it (C.S.); mongiardo@unicz.it (A.M.); jolesbt@hotmail.com (J.S.); 2 URT-CNR, Department of Medicine, Consiglio Nazionale delle Ricerche, 88100 Catanzaro, Italy

**Keywords:** TAVR, TAVI, low risk, STS, aortic stenosis, SAVR

## Abstract

Recently, two randomized trials, the PARTNER 3 and the Evolut Low Risk Trial, independently demonstrated that transcatheter aortic valve replacement (TAVR) is non-inferior to surgical aortic valve replacement (SAVR) for the treatment of severe aortic stenosis in patients at low surgical risk, paving the way to a progressive extension of clinical indications to TAVR. We designed a meta-analysis to compare TAVR versus SAVR in patients with severe aortic stenosis at low surgical risk. The study protocol was registered in PROSPERO (CRD42019131125). Randomized studies comparing one-year outcomes of TAVR or SAVR were searched for within Medline, Scholar and Scopus electronic databases. A total of three randomized studies were selected, including nearly 3000 patients. After one year, the risk of cardiovascular death was significantly lower with TAVR compared to SAVR (Risk Ratio (RR) = 0.56; 95% CI 0.33–0.95; *p* = 0.03). Conversely, no differences were observed between the groups for one-year all-cause mortality (RR = 0.67; 95% CI 0.42–1.07; *p* = 0.10). Among the secondary endpoints, patients undergoing TAVR have lower risk of new-onset of atrial fibrillation compared to SAVR (RR = 0.26; 95% CI 0.17–0.39; *p* < 0.00001), major bleeding (RR = 0.30; 95% CI 0.14–0.65; *p* < 0.002) and acute kidney injury stage II or III (RR = 0.28; 95% CI 0.14–0.58; *p* = 0.0005). Conversely, TAVR was associated to a higher risk of aortic regurgitation (RR = 3.96; 95% CI 1.31–11.99; *p* = 0.01) and permanent pacemaker implantation (RR = 3.47; 95% CI 1.33–9.07; *p* = 0.01) compared to SAVR. No differences were observed between the groups in the risks of stroke (RR= 0.71; 95% CI 0.41–1.25; *p* = 0.24), transient ischemic attack (TIA; RR = 0.98; 95% CI 0.53–1.83; *p* = 0.96), and MI (RR = 0.75; 95% CI 0.43–1.29; *p* = 0.29). In conclusion, the present meta-analysis, including three randomized studies and nearly 3000 patients with severe aortic stenosis at low surgical risk, shows that TAVR is associated with lower CV death compared to SAVR at one-year follow-up. Nevertheless, paravalvular aortic regurgitation and pacemaker implantation still represent two weak spots that should be solved.

## 1. Introduction

Transcatheter aortic valve replacement (TAVR) has been established as a standard of care for patients with severe aortic stenosis (AS) deemed at prohibitive or high surgical risk [1]. Of note, over the last years, its use has progressively increased, along with continuous improvements of devices and implantation techniques, to encompass patients at lower surgical risk [2,3]. Indeed, both the balloon-expandable as well as self-expandable devices were non-inferior to the surgical aortic valve replacement (SAVR) for short- and long-term outcomes in intermediate-risk patients [4] and are becoming a feasible alternative in appropriately selected low-risk patients. However, since SAVR has shown a low rate of mortality and stroke in these relatively young and healthy patients [5], some have hypothesized that the benefit of using TAVR may be futile over SAVR. Moreover, peri-procedural TAVR outcomes such as vascular access complications, conduction disturbances, bleeding and post-procedural paravalvular leak (PVL) after TAVR need further investigations in this large portion of patients [6]. Very recently, two randomized trials, the Safety and Effectiveness of the SAPIEN 3 Transcatheter Heart Valve in Low Risk Patients With Aortic Stenosis (PARTNER 3) trial [7], using the balloon-expandable valve, and the Evolut Low Risk Trial [8], using a self-expandable nitinol-frame valve, independently demonstrate that TAVR is non-inferior to SAVR in patients at low surgical risk. However, the non-inferiority design of these trials may be underpowered to detect statistical differences in hard clinical endpoints, as most were powered only for composite endpoints. Given this context, we have undertaken a systematic review and meta-analysis of the available evidence on TAVR to better characterize the safety and efficacy of the currently FDA-approved transfemoral TAVR in comparison with SAVR in patients with symptomatic aortic valve stenosis and at low operatory risk. 

## 2. Methods

### 2.1. Search Strategy and Study Selection

Published randomized trials comparing transcatheter to surgical aortic valve replacement were searched for within Medline, Scholar and Scopus electronic databases up to March 19th, 2019. The following syntax was used for the search: “transcatheter aortic valve replacement” OR “TAVR” OR “TAVI” AND “surgical aortic valve replacement” OR “SAVR” OR “SAVI” AND “low risk”. Time of publication and language were not limiting criteria for our analysis. All reports including the search terms were independently screened by two investigators for relevance and eligibility (A.P., S.S.). Additionally, references from relevant articles were also scanned for eligible studies. The authors discussed their evaluation and any disagreement was resolved through discussion and re-reading. All selected trials were thoroughly checked and classified by the author’s institution in order to avoid any effect from duplicity of data. The study protocol was registered in PROSPERO (CRD42019131125).

Studies were considered eligible if the following statements applied: (a) randomized clinical trials; (b) they involved a study population with aortic stenosis; (c) they compared TAVR versus SAVR; (d) they included mostly transfemoral TAVR (>95%); (e) they included patients at low risk (Society of Thoracic Surgeons (STS) score: ≤4); (f) follow-up length of 1 year; (g) they reported the following outcome data (all-cause mortality, cardiovascular death, myocardial infarction, stroke, transient ischemic attack, aortic regurgitation, new-onset atrial fibrillation, permanent pacemaker implantation, major and minor bleedings). Exclusion criteria were (just one was sufficient for study exclusion): duplicate publication, observational data.

### 2.2. Data Abstraction, Validity Assessment and Analysis

Baseline characteristics, as well as numbers of events, were extracted from the single studies, through careful scanning of the full article by two independent reviewers (A.P., S.S.). Divergences were resolved by consensus. In particular, the following data were abstracted: year of publication, location, number of study patients, study design, clinical outcome data (all-cause mortality, cardiovascular death, myocardial infarction, stroke, transient ischemic attack, aortic regurgitation, new-onset atrial fibrillation, permanent pacemaker implantation, major and minor bleedings) and baseline patients’ characteristics. Selection and data abstraction were performed according to the PRISMA statement [9]. The primary endpoint of this analysis was cardiovascular death. Further outcomes were: all-cause mortality, myocardial infarction (MI), stroke, transient ischemic attack (TIA), aortic regurgitation, new-onset atrial fibrillation, permanent pacemaker implantation, life-threatening or disabling bleeding and acute kidney disease (AKI) stage II or III. The quality of randomized trials included in the meta-analysis was appraised by using Cochrane methods (selection bias, performance bias, detection bias, attrition bias, reporting bias and other bias) as previously described [10].

### 2.3. Statistical Analysis

The summary measure used was the Risk Ratio (RR) with 95% confidence. The random-effects model was used, as previously described, to combine the collected values [11,12]. This model calculates a weighted average of the relative risks by incorporating within-study and between-study variations. Heterogeneity was assessed by means of the Cochrane Q test using a chi-squared function, with *p* < 0.10 considered significant for heterogeneity, as previously described [13]. Additionally, I^2^ values were calculated for the estimation of variation in weighted mean differences among studies attributable to heterogeneity. Power calculation of the meta-analysis was performed as described by Valentine et al. [14]. Small study effects were evaluated through graphical inspection of funnel plots, as already previously described [15]. Forest plots were used to graphically display the results of the meta-analysis, as already previously described [16]. Briefly, the measure of effect (RR) for every single study included (represented by a square) is plotted, together with confidence intervals, represented by horizontal lines. The area of each square is proportional to the study’s weight in the meta-analysis. The overall measure of effect is reported on the bottom line of the plot as a diamond, whose lateral ends indicate the confidence interval for the summary effect. Analyses were performed by means of RevMan 5.3.

## 3. Results

### 3.1. Search Results

**Figure 1:**
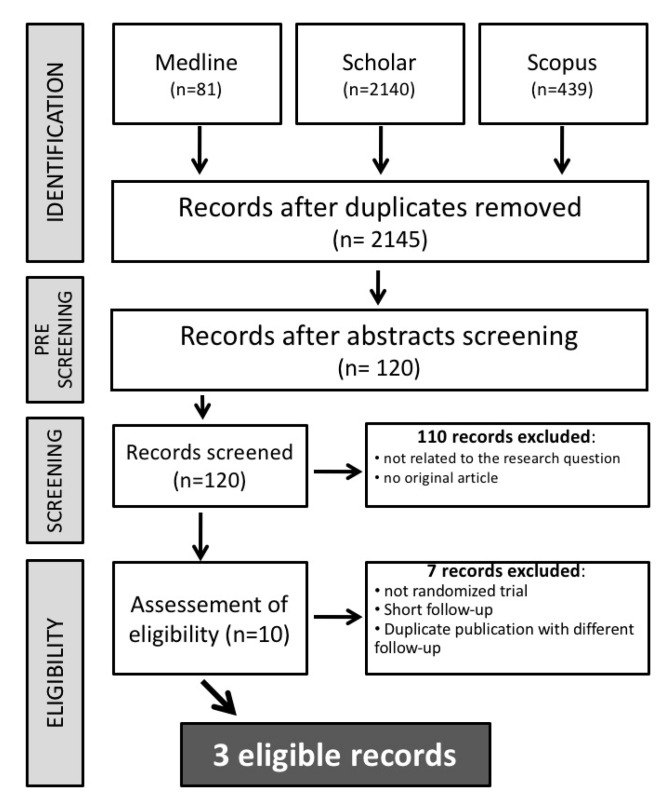
Study selection flow chart.

Our search retrieved a total of 2660 entries, which were reduced to 2145 studies after an initial pre-screening. A total of 110 studies were then excluded for one of the following reasons: (a) they were not related to our research question; (b) they were not original articles. In the assessment of eligibility, a further seven studies were excluded. Finally, a total of three studies were included [7,8,17]. The study selection procedure was reported in detail in Figure 1.

### 3.2. Study Characteristics

The main characteristics of the selected studies were reported in Table 1. Quality assessment revealed a high study quality (Supplementary Figure S1). The specific study designs made both patients’ and investigators’ blinding impossible. Endpoint assessment and data analysis was blinded in all included studies. A total of 2629 patients were included of which 1363 patients were randomized to TAVR and 1266 to SAVR. 

Baseline clinical and procedural characteristics across the trials are reported in Table 2. Across the studies, patients were predominantly male, one-fourth of patients had diabetes mellitus and less than 1% had creatinine level >2 mg/dL at presentation. The mean STS score was less than 3% across all the trials. In the TAVR arm, the most frequently implanted valve was a self-expandable valve which included Corevalve, Evolute R and Evolute PRO (Medtronic), whereas the remaining patients (*n* = 496) were treated with a balloon-expandable valve, Sapien 3 (Edwards). 

### 3.3. Study Outcomes

**Figure 2:**
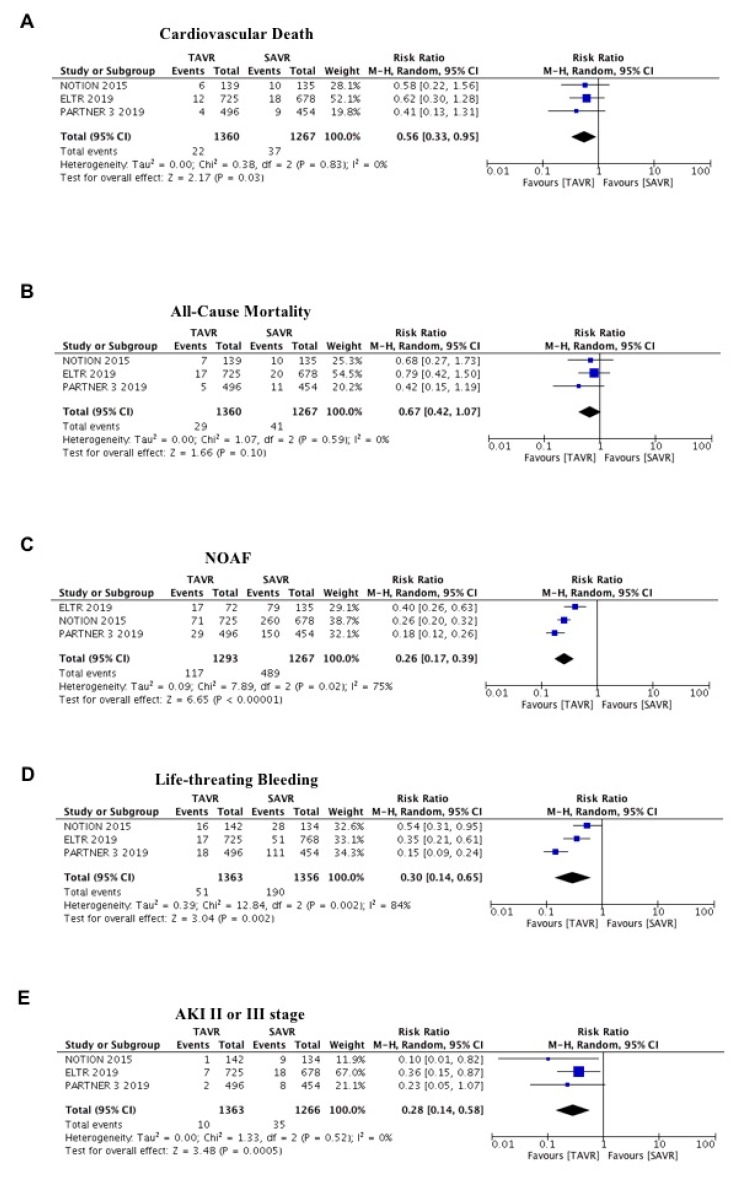
Meta-analysis of cardiovascular death, all-cause mortality, new-onset atrial fibrillation, life-threating bleeding, acute kidney injury II or III stage. (**A**) Forest plot and summary effect of the difference in the incidence of cardiovascular death, showing a significantly lower incidence in the TAVR arm (*p* = 0.03). (**B**) Forest plot and summary effect of the difference in the incidence of all-cause mortality showing no difference between transcatheter aortic valve replacement (TAVR) and surgical aortic valve replacement (SAVR; *p* = 0.10). (**C**) Forest plot and summary effect of the difference in the incidence of new-onset atrial fibrillation, showing a significantly lower incidence in the TAVR arm (*p* < 0.001). (**D**) Forest plot and summary effect of the difference in the incidence of life-threating bleeding, showing a significantly lower incidence in the TAVR arm (*p* < 0.002). (**E**) Forest plot and summary effect of the difference in the incidence of acute kidney injury II or III stage, showing a significantly lower incidence in the TAVR arm (*p* < 0.0005).

After one year, the risk of cardiovascular death was significantly lower with TAVR compared to SAVR (Risk Ratio (RR) = 0.56; 95% CI 0.33–0.95; *p* = 0.03; I^2^ = 0%; Figure 2A). Similarly, a trend risk reduction for one-year all-cause mortality was also observed in favor of TAVR (RR = 0.67; 95% CI 0.42–1.07; *p* = 0.10; I^2^ = 0%; Figure 2B). The effect was consistent also in fixed effect and no evidence of publication bias was found for this endpoint. Among the secondary endpoint, patients undergoing TAVR have a lower risk of new-onset of atrial fibrillation compared to SAVR (RR = 0.26; 95% CI 0.17–0.39; *p* < 0.00001; I^2^ = 75%; Figure 2C), major bleeding (RR = 0.30; 95% CI 0.14–0.65; *p* < 0.002; I^2^ = 84%; Figure 2D) and AKI stage II or III (RR = 0.28; 95% CI 0.14–0.58; *p* = 0.0005; I^2^ = 0%; Figure 2E). 

**Figure 3:**
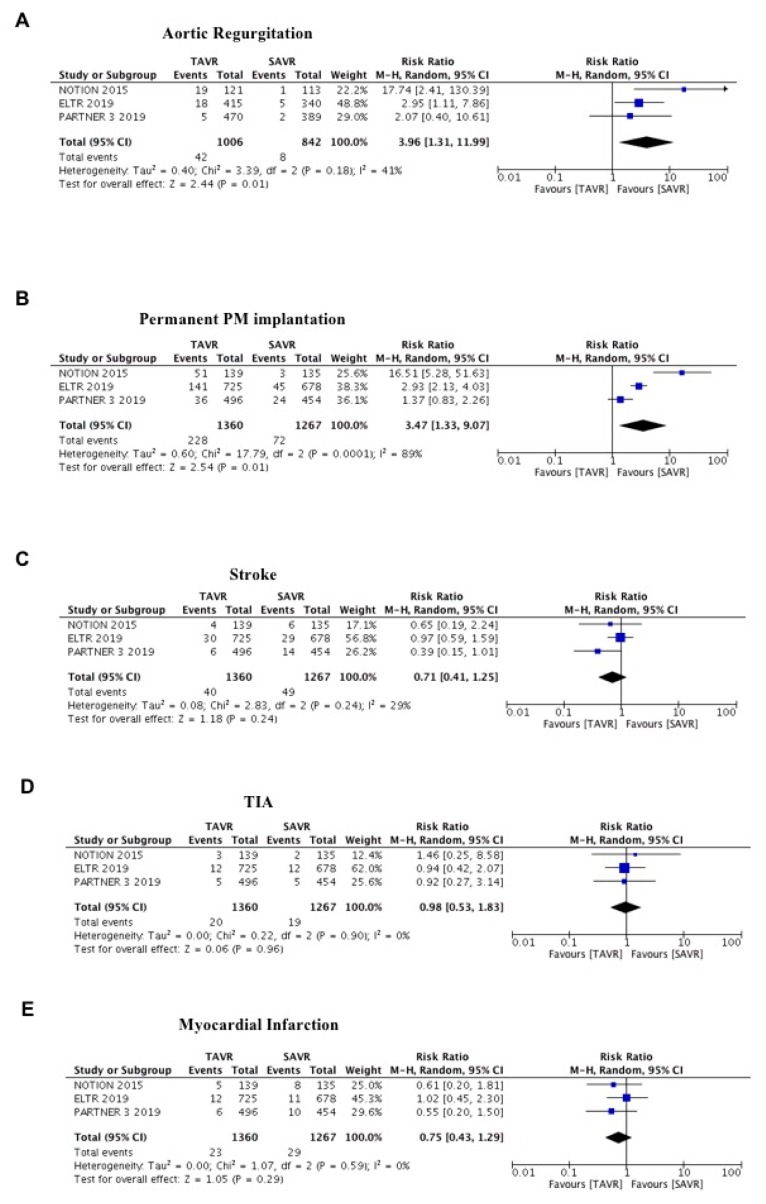
Meta-analysis of aortic regurgitation, permanent PM implantation, stroke, transient ischemic attack (TIA) and myocardial infarction. (**A**) Forest plot and summary effect of the difference in the incidence of aortic regurgitation, showing a significantly lower incidence in the SAVR arm (*p* = 0.01). (**B**) Forest plot and summary effect of the difference in the incidence of permanent PM implantation, showing a significantly lower incidence in the SAVR arm (*p* = 0.01). (**C**) Forest plot and summary effect of the difference in the incidence of stroke, showing no difference between TAVR and SAVR (*p* = 0.24). (**D**) Forest plot and summary effect of the difference in the incidence of TIA, showing no difference between TAVR and SAVR (*p* = 0.96). (**E**) Forest plot and summary effect of the difference in the incidence of myocardial infarction, showing no difference between TAVR and SAVR (*p* = 0.29).

Conversely, TAVR was associated to a higher risk of aortic regurgitation (RR = 3.96; 95% CI 1.31–11.99; *p* = 0.01; I^2^ = 41%; Figure 3A) and permanent pacemaker implantation (RR = 3.47; 95% CI 1.33–9.07; *p* = 0.01; I^2^ = 89%; Figure 3B) compared to SAVR. No differences were observed between the groups in the risks of stroke (RR = 0.71; 95% CI 0.41–1.25; *p* = 0.24; I^2^ = 29%; Figure 3C), TIA (RR = 0.98; 95% CI 0.53–1.83; *p* = 096; I^2^ = 0%; Figure 3D) and MI (RR = 0.75; 95% CI 0.43–1.29; *p* = 0.29; Figure 3E). The effect was consistent also in fixed effect and no evidence of publication bias was found for this endpoint for all the secondary outcomes. 

## 4. Discussion

We performed a meta-analysis with only randomized studies comparing a one-year outcome after treatment of severe aortic stenosis with TAVR or SAVR in patients at low surgical risk. 

Recently, several meta-analyses showed promising results of TAVR [18,19,20]. However, there are some differences with our meta-analysis. Kheiri et al. performed a meta-analysis of patients at low risk, but including transapical TAVR [21] and a post-hoc analysis of the SURTAVI trial [22]. Similarly, Kolte and colleagues also included in their work the post-hoc analysis of the SURTAVI trial finding a significant difference in the rate of all-cause death [20]. Conversely, we found only a nonsignificant difference in such hard clinical end-points. A recently updated meta-analysis [18] of RCTs including all surgical risk categories had reported a reduction in all-cause mortality up to two years of TAVR irrespective of baseline surgical risk. However, in the subgroup at low surgical risk, they had reported only all-cause mortality outcomes with a dishomogenous follow-up. Hence, different TAVR access approaches were performed in most of the studies. In our meta-analysis, we included only studies with >95% of transfemoral access-site since current data and expertise strongly favor the femoral artery as the preferred and most widespread access route for TAVR.

In the present meta-analysis, including three randomized studies and nearly 3000 patients, we found a superiority of TAVR against SAVR for cardiovascular death (primary endpoint) at the one-year follow-up (Figure 2A). Interestingly, results on this hard clinical endpoint were strongly homogeneous across individual studies and could be explained by several factors. Patients undergoing TAVR had less: acute kidney injury (Figure 2E); new-onset atrial fibrillation (Figure 2C); major bleedings (Figure 2D). These factors could have a strong impact on mortality, also in the long-term.

The second key finding of our meta-analysis is a significant reduction in the risk of AKI in patients undergoing TAVI compared with SAVR. Several studies have shown that AKI is a serious complication after both TAVR and SAVR. Adams and colleagues reported a lower incidence with TAVR compared to SAVR (6.0% vs. 15.1%; *p* < 0.001) [23]. Similarly, Bagur et al. showed that 9% suffered from AKI after TAVR, whereas SAVR was associated with an incidence of AKI in 26% [24]. These results could be of impact, in fact, AKI was associated with an increased risk of 30-day and long-term (up to seven years) mortality (42.3% versus 22.7% for seven-year mortality; HR 1.71 (95% CI 1.30–2.25)) [25].

The third key finding of our meta-analysis is a significant reduction in the risk of new-onset atrial fibrillation (NOAF) in patients undergoing TAVI compared with SAVR. New-onset atrial fibrillation (NOAF) has emerged in the last few years as a potential prognostic factor in patients undergoing TAVR [12]. NOAF after TAVR could be detrimental due to atrio-ventricular dyssynchrony resulting in reduced cardiac output and increased filling pressures. In addition, NOAF could be responsible for fatal cerebrovascular events. Recently, Gargiulo et al. performed a meta-analysis of eight studies encompassing 4959 patients to investigate the role of NOAF as a potential prognostic factor in patients undergoing TAVR. Interestingly, they found a borderline increase of 30-day and a significant increase in one-year all-cause death in the NOAF group compared with those in sinus rhythm [26].

The fourth key finding of our meta-analysis is a significant reduction in the risk of NOAF in patients undergoing TAVI compared with SAVR. The impact of bleeding on hard clinical endpoints in patients undergoing TAVR was already discussed by Piccolo et al. [27]. Among patients with severe aortic stenosis undergoing TAVR, both access-site and non-access-site bleeding were independently associated with an increased risk for mortality.

Finally, all these three factors (bleedings, AKI, NOAF) for the reasons mentioned above, could explain, at least in part, the superiority of TAVR against SAVR for cardiovascular death, also in a population at low surgical risk at one-year follow-up.

Cerebral embolization is a common complication leading to stroke after TAVR and SAVR [28]. In this meta-analysis, we found no difference in stroke and TIA rates between the groups. These results are in line with the results of single studies [7,8,17].

However, some concerns should be raised, and some limitations should be mentioned about TAVR: (1) paravalvular aortic regurgitation; (2) major incidence of pacemaker implantation; (3) durability at longer follow-up. 

Paravalvular aortic regurgitation and the need for permanent pacemaker implantation have historically been the limit of TAVR compared with SAVR. Nevertheless, advances in TAVR technology, along with operator experience, precise valve sizing and implantation technique, may further reduce the associated pacemaker implantations and paravalvular aortic regurgitation risks. However, in a recent real-world study of TAVR among lower surgical risk patients, promising rates of 30-day moderate-to-severe paravalvular leak (0.5%) and permanent pacemaker implantation (6.5%) were reported [29].

Finally, the question of durability has been an Achilles heel of TAVR. Some recent data seem to suggest similar longevity between transcatheter and surgical tissue valves out to five to seven years. A post-hoc analysis wherein investigators looked for valve dysfunction and failure in the NOTION trial showed that bioprosthetic valve dysfunction was numerically lower in TAVR group over five years (55.4% versus 65.2%, *p* = 0.10) [30] compared to SAVR. However, to date, it is not clear if that is good enough to get TAVR into younger patients.

## 5. Limitations

As for any meta-analysis, some limitations should be acknowledged that are related to: (1) different definitions in the studies for different endpoints; (2) differences in the baseline characteristics between the studies; (3) not all the outcomes are reported in the studies; (4) short-term follow-up.

## 6. Conclusions

The present meta-analysis, including three randomized studies and nearly 3000 patients with severe aortic stenosis at low surgical risk, shows that TAVR is associated with lower CV death compared to SAVR at 1-year follow-up. Nevertheless, paravalvular aortic regurgitation and pacemaker implantation still represent two weak spots that should be solved. 

## Figures and Tables

**Table 1:** Characteristics and endpoint definitions of included randomized trials.

Study	Year	Location	N	Study Design	Primary Endpoint	Mortality Reported	Valve Type	Randomization	Follow-Up (Years)
NOTION	2015	Multicenter	280	RCT	Death from any cause, stroke, or myocardial infarction	Yes	CoreValve (Medtronic)	TAVR vs. SAVR	1
ELRT	2019	Multicenter	1403	RCT	Death from any cause or disabling stroke	Yes	CoreValve, Evolut R, or Evolut PRO (Medtronic)	TAVR vs. SAVR	1
PARTNER 3	2019	Multicenter	950	RCT	death from any cause, stroke, or rehospitalization	Yes	Sapien 3 (Edwards)	TAVR vs. SAVR	1

Abbreviations: RCT = randomized clinical trials; TAVR = transcatheter aortic valve replacement; SAVR = surgical aortic valve replacement.

**Table 2:** Patient’s characteristics.

	NOTION 2015		ELRT 2019		PARTNER 3 2019	
	TAVR	SAVR	TAVR	SAVR	TAVR	SAVR
N of patients, *n*	145	135	725	678	496	454
Age, yrs	79.2	79	74.1	73.6	73.3	73.6
Male, %	53.8	52.6	64	66.2	67.5	71.1
Creatinine > 2 mg/dL, %	1.4	0.7	0.4	0.1	0.2	0.2
Peripheral vascular disease, %	4.1	6.7	7.5	8.3	6.9	7.3
Diabetes, %	17.9	20.7	31.4	30.5	31.2	30.2
Chronic lung disease, %	11.7	11.9	15	18	5.1	6.2
Prior Stroke, %	16.6	16.3	10.2	11.8	3.4	5.1
Prior MI, %	5.5	4.4	6.6	4.9	5.7	5.8
Prior AF, %	27.8	25.6	15.4	14.5	15.7	18.8
STS score, %	2.9	3.1	1.9	1.9	1.9	1.9

Yrs = years; MI = myocardial infarction; AF = atrial fibrillation; STS = Society of Thoracic Surgeons.

## Supplementary Materials

The following are available online at https://www.mdpi.com/2077-0383/9/2/439/s1, Figure S1: Quality assessment.
