# Supplementary material for: Transcatheter Versus Surgical Aortic Valve Replacement in Low-Risk Patients for the Treatment of Severe Aortic Stenosis
Source: J Clin Med. 2020 Feb 6;9(2):439. doi: 10.3390/jcm9020439 (PMC7074202; doi:10.3390/jcm9020439)
Supplement: Supplementary file 1 [file jcm-09-00439-s001.zip › jcm-684184-supplementary.pptx]

## Slide 1
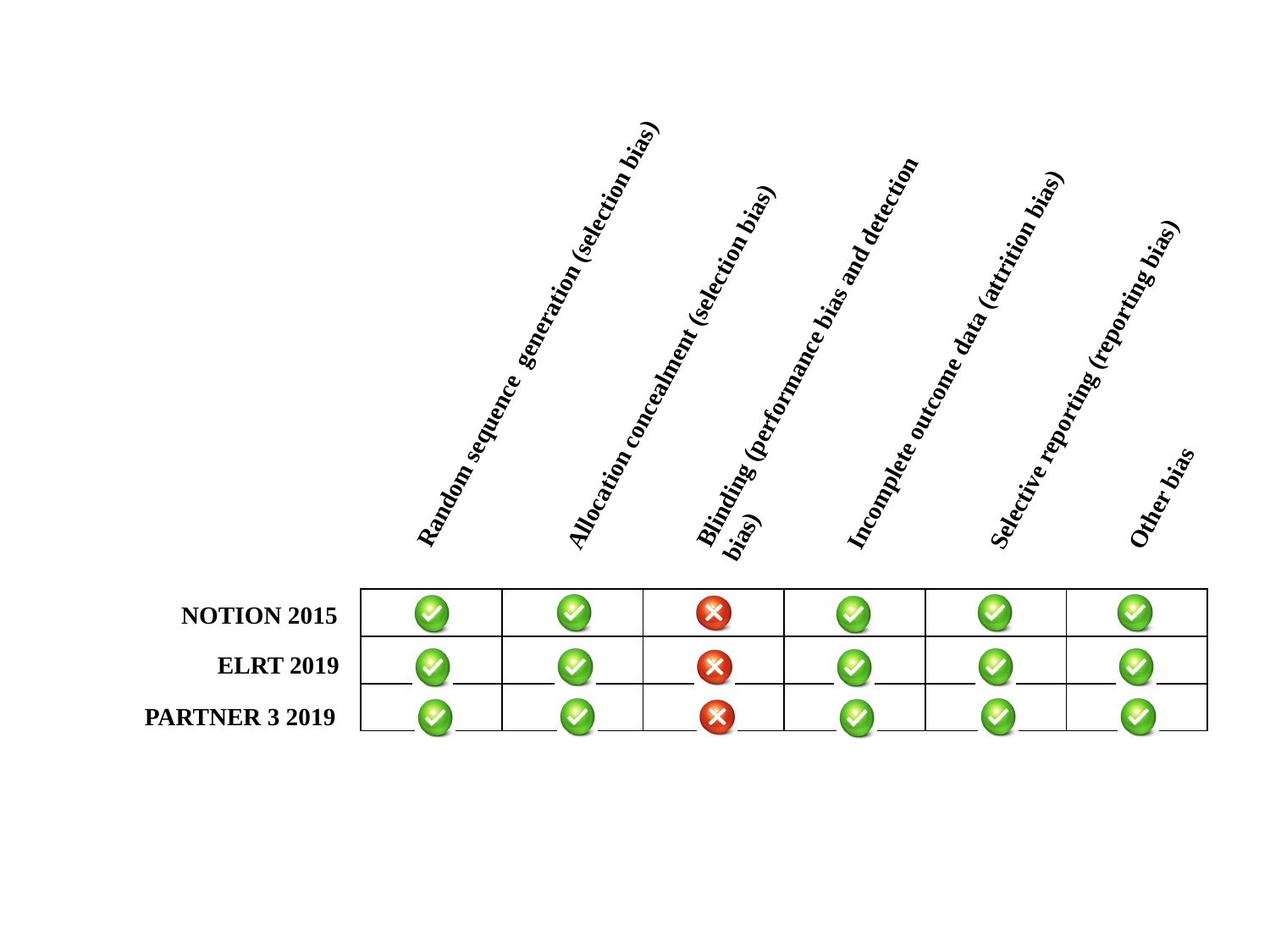

Random sequence generation (selection bias)
Blinding (performance bias and detection bias)
Allocation concealment (selection bias)
Incomplete outcome data (attrition bias)
Selective reporting (reporting bias)
Other bias
| | | | | | |
| --- | --- | --- | --- | --- | --- |
| | | | | | |
| | | | | | |
NOTION 2015
ELRT 2019
PARTNER 3 2019
